# Deep learning-based target spraying control of weeds in wheat fields at tillering stage

**DOI:** 10.3389/fpls.2025.1540722

**Published:** 2025-03-27

**Authors:** Haiying Wang, Yu Chen, Shuo Zhang, Peijie Guo, Yuxiang Chen, Guangrui Hu, Yuxuan Ma

**Affiliations:** ^1^ College of Mechanical and Electronic Engineering, Northwest A&F University, Yangling, China; ^2^ School of Design, Xi’an Technological University, Xian, China

**Keywords:** weed identification, weed distribution determination, hysteresis property, target spraying, deep learning

## Abstract

In this study, a target spraying decision and hysteresis algorithm is designed in conjunction with deep learning, which is deployed on a testbed for validation. The overall scheme of the target spraying control system is first proposed. Then YOLOv5s is lightweighted and improved. Based on this, a target spraying decision and hysteresis algorithm is designed, so that the target spraying system can precisely control the solenoid valve and differentiate spraying according to the distribution of weeds in different areas, and at the same time, successfully solve the operation hysteresis problem between the hardware. Finally, the algorithm was deployed on a testbed and simulated weeds and simulated tillering wheat were selected for bench experiments. Experiments on a dataset of realistic scenarios show that the improved model reduces the GFLOPs (computational complexity) and size by 52.2% and 42.4%, respectively, with mAP and F1 of 91.4% and 85.3%, which is an improvement of 0.2% and 0.8%, respectively, compared to the original model. The results of bench experiments showed that the spraying rate under the speed intervals of 0.3-0.4m/s, 0.4-0.5m/s and 0.5-0.6m/s reached 99.8%, 98.2% and 95.7%, respectively. Therefore, the algorithm can provide excellent spraying accuracy performance for the target spraying system, thus laying a theoretical foundation for the practical application of target spraying.

## 1 Introduction

Wheat stands as a globally cultivated cereal crop, encompassing over 22.067 billion hectares planted annually across a spectrum of climatic conditions and diverse geographic locales (Shiferaw et al., 2013), surpassing all other cultivated food crops in scale (Khan et al., 2024). Despite its vast reach, the menace of weeds looms large, imperiling optimal crop yields by vying with crops for vital resources like water, nutrients, and sunlight (Horvath et al., 2023), leading to yield reductions in wheat grains of up to 24% (Jabran et al., 2017). Herbicides have emerged as pivotal agents fostering crop yield proliferation since the latter half of the 20th century (Cassman, 1999).Amidst the efficiency of herbicides in directly combating weeds, farmers frequently resort to substantial herbicide applications to safeguard against yield decline and compromised agricultural product quality. Nonetheless, the extensive use of agrochemicals exacts a toll on environmental integrity, biodiversity, and human well-being (Sharma et al., 2019). In a departure from indiscriminate broadcast spraying, targeted spraying techniques leverage the discernment of target size and location within fields to regulate the activation of corresponding nozzles, offering a potent solution to challenges like herbicide excess and fluid wastage (Yu et al., 2019).

In recent years, the application of machine vision technology in agricultural production has been widely studied in all aspects of agricultural production (Lv et al., 2024). Traditional machine vision methods usually use manually designed feature extraction methods, which are difficult to capture the complex features of weeds and lack flexibility and generalization. Deep learning (DL) techniques are an emerging artificial intelligence approach that allows for accurate and fast object detection by automatically learning and understanding low-level to high-level image features based on a conceptual hierarchy (LeCun et al., 2015). Recent developments in deep learning have been shown to be effective in a variety of crop management operations (Brown et al., 2008). Modi et al. (2023) used a deep learning model to identify weeds in sugarcane fields and found that the DarkNet53 model outperformed the other models by showing high accuracy and F1 scores in identifying weeds in sugarcane crops. Yuan et al. (2023) constructed a WeedyRice5 target detection model based on the YOLOv5s method for detecting weeds in rice. Shao et al. (2023) proposed an improved deep learning model, GTCBS-YOLOv5s, utilizing Ghost, C3Trans, and CBAM modules that improved weed feature extraction level. Zhu H. et al. (2024) connected a lightweight attention module to the deep network of YOLOX-Darknet, which weakened the channel noise effect of residual computation and made the detection model more efficient. Deng et al. (2024) INet-based model improved the accuracy of YOLOX and YOLOv8 compared to the original model by respectively 1.4% and 3.3%, respectively. Rai et al. (2024) optimized and reconstructed the YOLO-Spot model constructed based on YOLOv7-tiny to identify weeds in crop plants, and the optimized model could use a smaller number of training parameters to identify weeds. Alrowais et al. (2022) combined the HLBODL-WDSA model combined with YOLOv5 for weed detection, where the HLBO algorithm was used as a hyper-parameter optimizer to efficiently classify weeds using a Kernel Extreme Learning Machine (KELM) model. Rahman et al. (2023) used YOLOv5, RetinaNet, EfficientDet, Fast RCNN, and Faster RCNN, to 13 weed detection models were constructed by transferring pre-trained target detection models to the weed dataset, and the data were enhanced by geometric and color transformations. Although the YOLO algorithm has excellent recognition results, it is difficult to be directly deployed on in-vehicle devices with limited computational power due to its high requirements on device arithmetic. Improvements that reduce the computational complexity of the model and increase its accuracy are particularly important given the need for high accuracy in weed detection.

In the area of targeted spraying, Upadhyay et al. (2024) designed and developed a machine vision-based spraying system specifically designed to identify weeds and enable precision spraying. The spraying platform employs a deep-learning YOLOv4 model to accurately recognize a wide range of weeds, thereby facilitating targeted spraying applications. The system is equipped with a FLIR RGB camera for real-time image acquisition, while an Nvidia Jetson AGX Orin is used as an edge device to deploy the deep learning model for weed detection. The Nvidia Jetson pin is used to activate a relay for precise on/off control of a solenoid valve for spot spraying. In the indoor experiment, the spray system achieved an average effective spray rate of 93.33% with 100% accuracy, while the recall rate was 92.8%. In contrast, the field experiment had a slightly lower average effective spray rate of 90.6%, but still maintained an accuracy rate of 95.5% and a recall rate of 89.47%. Sunil et al. (2024) developed a weed spraying robotic platform using the NVIDIA Jetson embedded device as the control and processing unit and tested it under laboratory and field conditions. Fan et al. (2023) developed a deep-learning-based seedling weed detection and on-target spraying robotic system for cotton fields, which is capable of field weed detection and herbicide spraying, laying the foundation for targeted spraying in weed control. However, target spraying robots are expensive and cannot be popularized in large-scale field crops due to their high R&D costs and low operational efficiency. Compared to target spraying robots, target spraying systems mounted on sprayers are less expensive, and target spraying systems can be deployed on existing sprayers relatively easily without major modification or redesign of existing sprayers., the spraying volume of the sprayer equipped with the on-target spraying system was reduced by 40% and the ground deposition was reduced by 41%, and the sprayer of this experiment was operated at a constant speed. Li et al. (2022a) designed a grid decision algorithm for the switching of solenoid valves group to convert the single weed position information in the image into the opening and closing control information of solenoid valves. The current target spraying system mainly focuses on the identification and localization of single plants, while effective treatment solutions in the case of complex plant distribution still need further research. Meanwhile, the hysteresis between target spraying hardware remains a major challenge in target spraying.

The research content and contributions of this paper include: first, the overall scheme of the target spraying control system is proposed. Then, Yolov5s is lightened and improved to make the model balanced in terms of weed recognition accuracy and computational complexity. Based on this, a target spraying decision-making and hysteresis algorithm was designed to control the solenoid valve to differentiate spraying for weeds with different distributions after completing the weed identification, and effectively solved the hysteresis problem existing between spraying hardware. A target spraying control system test bed was developed, and the integrated algorithm was deployed on the test bed for target spraying experiments. The experiment shows that the algorithm has high accuracy in spraying weeds with different distribution and can effectively reduce the waste of herbicides.

## 2 Materials and methods

### 2.1 Overall program design

**Figure 1:**
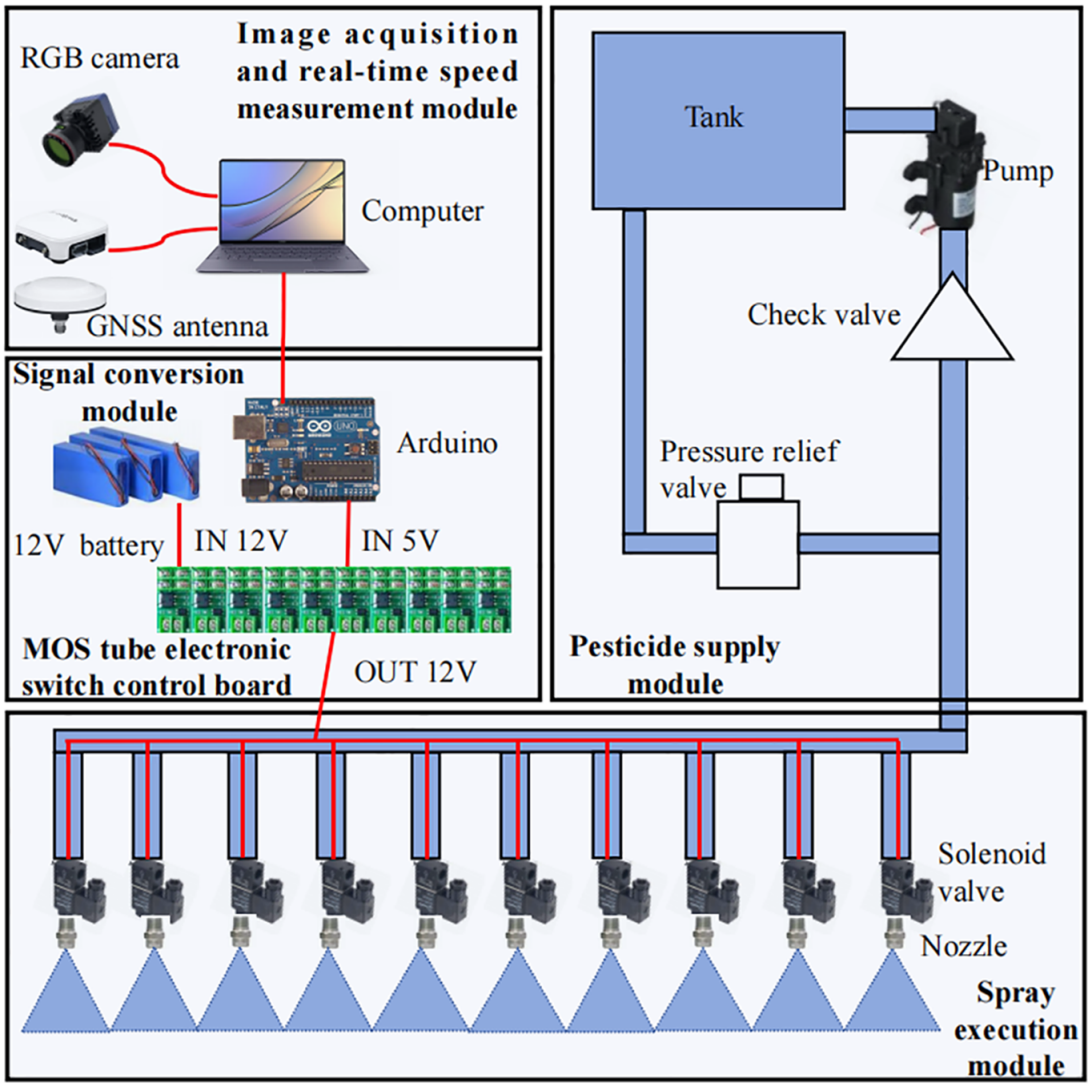
Control principle diagram of single pair target spraying system.

In order to achieve precise weed control in wheat fields at tillering stage, this study developed a pair-target control system that can detect and spray multiple weeds randomly distributed in wheat fields in real time and on-target, so that the utilization rate of herbicides is improved. A single target spraying control system includes an image acquisition and real-time speed measurement module, a signal conversion module, a spray execution module, and a herbicide supply module. The system control principle is shown in **Figure 1**.

The image acquisition and real-time speed measurement module consists of an RGB camera, a GNSS antenna and a computer. The RGB camera is responsible for capturing images of weeds in the wheat field. The sprayer operation can use dual GNSS antennas for differential speed measurement, and the frequency of the feedback speed is 10Hz.The computer is responsible for processing the image information from the RGB camera and the speed data from the GNSS antenna, and sends target spraying commands to the Arduino. The RGB camera is a W200 model from Jierui Microcomputer Technology Co., Ltd (Shenzhen, China), with a 2.9MM distortion-free 130-degree wide-angle and 200W pixels. The frame rate is 60 frames per second, the power is 2W, the working voltage is 5V, and the working current is 120mA~220mA.

The signal conversion module consists of an Arduino, a 12V battery and ten MOS electronic switch control boards. The ten pins of the Arduino are connected to the signal terminals of the ten MOS electronic switch control boards. The Arduino is responsible for converting the target spraying commands sent by the computer into the high and low levels of the pins. Since the high level of the Arduino pins is 5V, it cannot directly drive the 12V solenoid valve to work. Therefore, by connecting a 12V battery to the input of the MOS electronic switch control board, when the level of the signal terminal is high, the output of the MOS electronic switch control board will output 12V current to drive the solenoid valve to open; when the signal terminal is low, the solenoid valve is closed.

The spray actuator module consists of 10 solenoid valves and 10 spray nozzles. The positive and negative poles of each solenoid valve are connected to the output of the respective MOS tube electronic switch control board. The solenoid valve model is HL22-02 of Honglian Control Flow Technology Development Co., Ltd (Ningbo, China), with a pressure of 8MPa, G1/4 (2 points) interface, and a voltage of DC12V. A stainless steel fan nozzle, model 3004, with a spray angle of 30 degrees and an aperture of 1.3mm, from Geo Industrial Spray Equipment Co. The flow rate of the nozzle was 1.2 L/min at an operating pressure of 0.8 MPa. The maximum diameter of weeds in a wheat field at the tillering stage is usually no more than 10 cm, and because multiple weeds are often clustered in a wheat field, the diameter of the patches formed can be 20 cm or even larger. Therefore, at a height of 42cm, the spray coverage length using a 30° nozzle is 23cm, with a 1.5cm spray overlap area on each side of the nozzle to ensure that leakage is prevented. The nozzles are fan-shaped and are connected to a solenoid valve.

The herbicide supply module consists of a water tank, a pump, a check valve and a safety valve. The pressure of the safety valve is set to 0.8Mpa. When the pressure of the pipeline exceeds 0.8Mpa, the safety valve will automatically release the pressure and discharge the excess liquid into the water tank to protect the pipeline. The one-way valve is used to prevent the liquid from flowing back. When expanding multiple pairs of target spraying control systems, it is possible to realize that multiple spraying execution modules share one herbicide supply module according to the working parameters of the pump.

**Figure 2:**
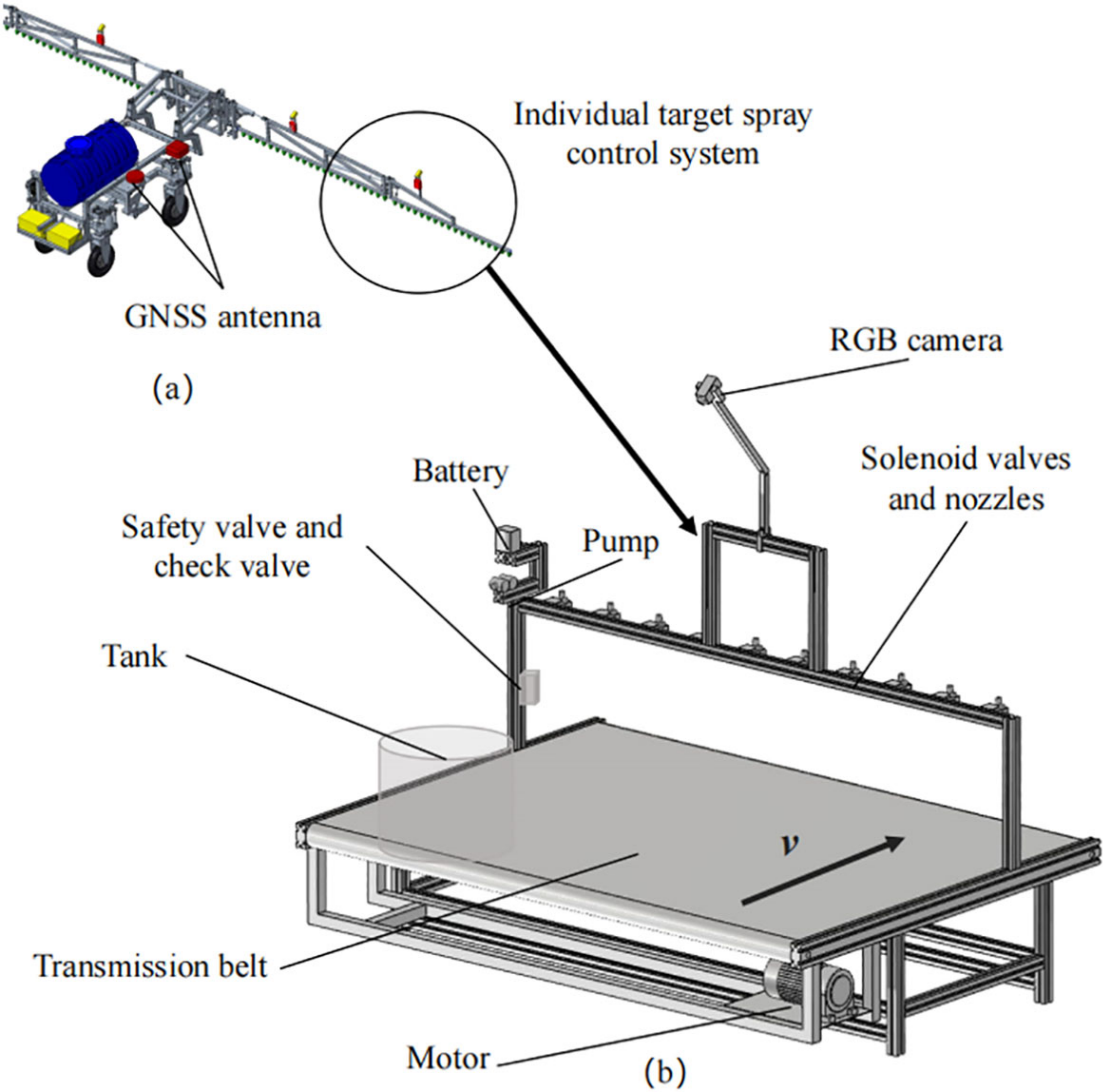
Structural schematic diagram: **(a)** a schematic diagram of the structure of a sprayer equipped with a plurality of pairs of target spray control systems, and **(b)** a schematic diagram of the structure of a single target spray control system test bed.

Multiple pairs of target spray control systems can be assembled on the spraying machine, as shown in **Figure 2a**. The structure of a single counter-target spray system test bed is shown in **Figure 2b**, including an RGB camera mounted at a height of 1.5 m from the upper surface of the conveyor belt, with a shooting area width of 2 m. The camera is mounted at a height of 1.5 m from the upper surface of the conveyor belt. Ten solenoid valves were mounted on the spray bar below the camera, and the height of the spray nozzles from the upper surface of the conveyor belt was 42 cm, and the spray coverage of each nozzle was about 24 cm. Simulated weeds and simulated wheat were placed on the conveyor belt, and the motor drove the simulated weeds and simulated wheat on the conveyor belt to move in the direction v in **Figure 2** to simulate the operation of the sprayer in the field.

### 2.2 Weed data set collection and processing

**Figure 3:**
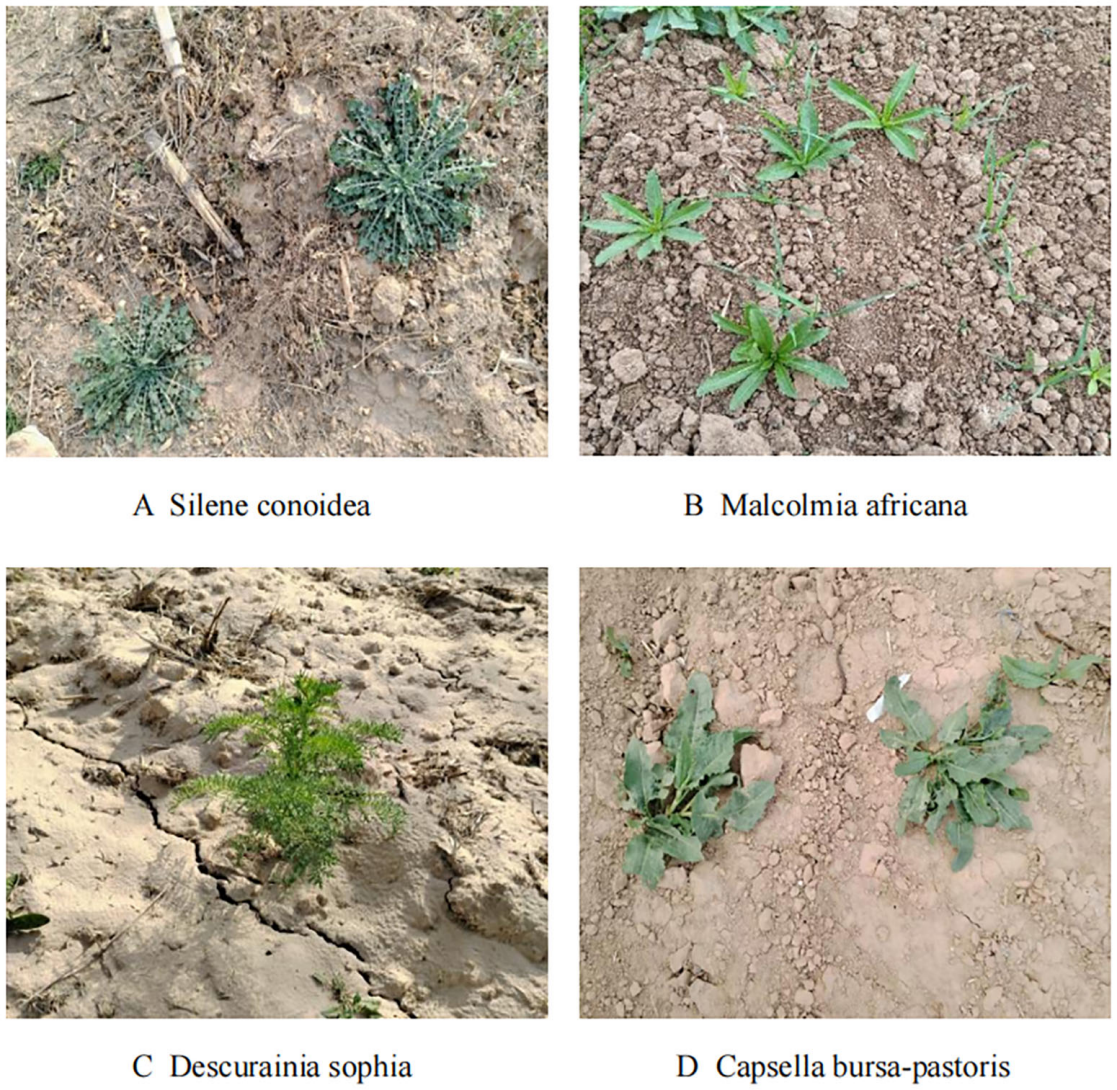
Example of weeds in wheat field at tillering stage **(A)** Silene conoidea **(B)** Malcomia africana **(C)** Descurainia sophia **(D)** Capsella bursa-pastoris.

Within Shaanxi Province, weeds in wheat fields are mainly dominated by Cruciferae and Gramineae, and some of the more damaging weeds include Silene conoidea, Malcolmia africana, Descurainia sophia, and Capsella bursa-pastoris. In this study, crop and weed growth in wheat field fields at the tillering stage was collected in Yangling District and Heyang County, Shaanxi Province, and the data were collected at the tillering stage of wheat (when the leaves grew to four to six). Part of the dataset is shown in **Figure 3**.

In order to ensure the diversity of the produced dataset, 1012 images in the environments of complex, dense, uneven light and shade backgrounds were selected as the raw data, respectively. And Matlab software was used to add pretzel noise and Gaussian noise to some of the datasets for image processing, which helps to improve the robustness of the algorithm by introducing noise to reduce the clarity of the image. The dataset images are expanded to 1600 images by image enhancement. The dataset types are shown in **Table 1**.

**Table 1:** Types of data sets.

Data types	Number of data
Complex environmental context	236
Dense distribution	212
Uneven lighting	296
Occlusion	268
Pepper noise image enhancement	283
Gaussian noise image enhancement	305

### 2.3 Improved weed detection model based on Yolov5s

In terms of lightweight deep learning models for identifying weeds, there are mainly single-stage networks and two-stage networks, and the average accuracy mAP of single-stage networks such as SSD, RetinaNet, EfficientDet, CenterNe, etc., in identifying weeds needs to be further improved (Saleem et al., 2022), and the RFCN and Faster RCNN as represented by RFCN, Faster RCNN need to be further optimized in terms of computational complexity and inference time (Fan et al., 2023). And Yolo series is an algorithm for fast object recognition using neural networks. The Yolo series algorithms are a fast object recognition method based on neural networks, and their versions are Yolov9, Yolov8, Yolov7, Yolov5, Yolov4, and Yolov3 according to the time of their release. However, the newer is not always the better when choosing the version of a model. Among the models in the same class, Yolov9s (26.7 GFLOPs) and Yolov8s (28.6 GFLOPs) excel in recognition accuracy, but their GFLOPs (computational complexity) are much higher than those of Yolov5s (15.8 GFLOPs). Therefore, when choosing a version of the Yolo algorithm, it is necessary to make a judgment based on a specific recognition scenario.Yolov5 contains five different versions: Yolov5n, Yolov5s, Yolov5m, Yolov5l, and Yolov5x.The main differences between these versions are the depth and width of the model, which is also known as its complexity and performance. The smaller the model, the lower the computational complexity and the faster it runs, but the detection accuracy decreases. For weed target recognition in the field, the model should have the characteristics of high accuracy, low computational complexity and fast recognition.Yolov5s has the advantages of fast speed and high accuracy and shows good processing ability in the case of overlapping targets (Zhu A. et al., 2024), but Yolov5s is not sufficiently accurate in recognizing some of the small weeds. To further reduce the computational complexity of Yolov5s, which can be deployed on vehicle-mounted devices with limited computational power, this study improves Yolov5s by lightweighting and enhancing its accuracy.

#### 2.3.1 Lightweight backbone network construction

Yolov5s uses C3 and Conv as the backbone feature extraction network, while an image after neural network feature extraction generates many feature maps, some of these feature maps are very similar, increasing the number of parameters and making it slower. While Ghost Module (Han et al., 2020) can perform a simple operation on one of the feature maps to generate more similar feature maps. In order to be able to ensure efficient operation and reduce repeated calculations, therefore, this study uses the lightweight model Ghost Module to construct the backbone network.

#### 2.3.2 Neck network construction

In Yolov5s, the neck is the part that connects the backbone to the detection and is responsible for feature fusion and processing. In the scenario of real-time weed detection, traditional large-scale models are difficult to meet the requirements of real-time detection, while lightweight models constructed from a large number of depth-separable convolutional layers are also unable to achieve sufficient accuracy while ensuring real-time performance. Therefore, this study uses a special lightweight convolution technique GSConv (Li et al., 2022b). GSConv first downsamples the input, then uses DWConv deep convolution on the downsampled result, and splices the downsampled result with the deep convolution, and finally performs a shuffle operation and outputs it, which reduces the parameters while as much as possible The connection between the neck and the backbone is preserved, so that the feature map no longer needs to be transformed when it is transmitted to the neck, and there are fewer redundant repetitions. The cross-level network module VoVGSCSP is designed based on GSConv using the one-time aggregation method, and combined with GSConv to form the Slim-Neck architecture.

#### 2.3.3 Self-attention mechanism

Most of the existing attention mechanisms use additional sub-networks to generate the attention weights, which increases the number of parameters of the neural network, so this study uses a self-attention mechanism SimAM (Yang et al., 2021), which does not need to add parameters to the original network. Its input data size is *c×h×w*, the 3D weights of the feature maps are inferred by the energy function, and the weights normalized by the Sigmoid function are multiplied with the original feature maps to get the output feature maps of the boosted features, so that the model focuses more on the important parts and improves the performance of detecting the target.

#### 2.3.4 Improved model

**Figure 4:**
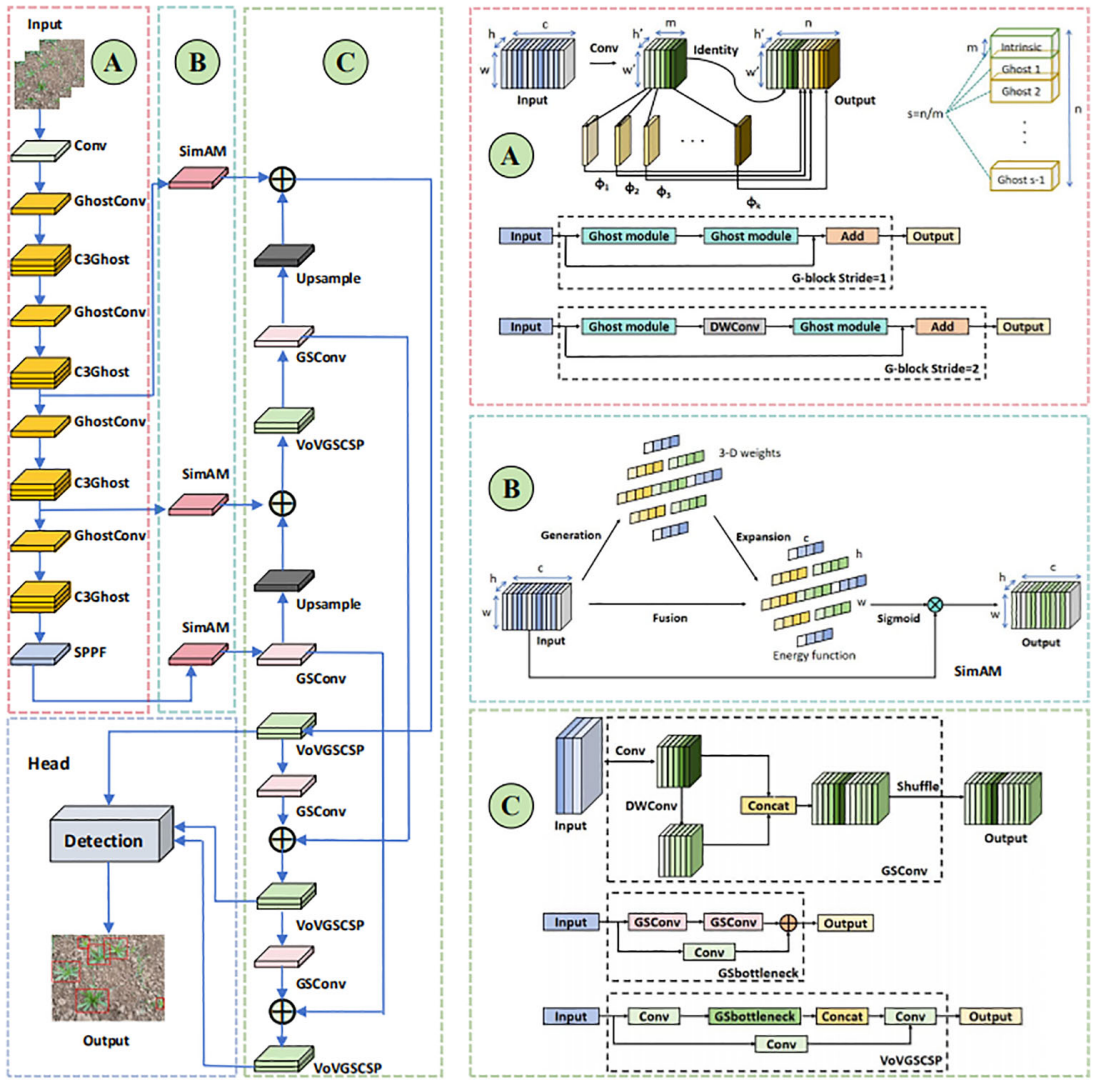
**(A–D)** Yolov5-SGS model network structure.

In this study, we propose a lightweight Yolov5-SGS model. First, we replace C3 and Conv in Yolov5s feature extraction backbone network with the lightweight C3Ghost and GhostConv. Then, C3 and Conv in the original neck network are replaced with the more lightweight and efficient GSConv and VoVGSCSP, respectively, to speed up the feature fusion and processing. Finally, the SimAM self-attention module is added to each of the three outputs connected to the feature extraction backbone network and the neck network to process the outputs of the forward transfer layer, increase the distinction between weeds and complex backgrounds, and improve the weed feature salience. The improved model network structure is shown in **Figure 4**.

### 2.4 Target spraying decision and hysteresis algorithms

In the target spraying system, the key to controlling the spray nozzle for target spraying lies in controlling the opening and closing moments of the solenoid valves and their durations. Li He et al. (2022) designed a grid decision algorithm for the switching of solenoid valves, which converts the position information of a single weed in the image into the opening and closing control information of the solenoid valves. For a single weed, the solenoid valve can be opened and closed normally in the response frequency to complete the target spraying. However, the distribution of weeds in the wheat field is random, for the distance is very close to the multi-plant weeds, the solenoid valve can not respond very quickly, which will lead to the liquid can not spray the neighboring weeds. Therefore, the opening and closing frequency of the solenoid valve determines the minimum distance between multiple weeds that can be sprayed, and the length of the weeds determines the duration of the solenoid valve opening. In the butt-target spraying control system, there is a hardware system delay problem from the completion of the butt-target spraying decision to the control of the solenoid valve spraying, as well as an unavoidable hysteresis distance between the area where the camera recognizes the weeds and the spray nozzle. We have investigated the above problems and designed a target spraying decision and hysteresis algorithm.

#### 2.4.1 Algorithm for decision making on target spraying

**Figure 5:**
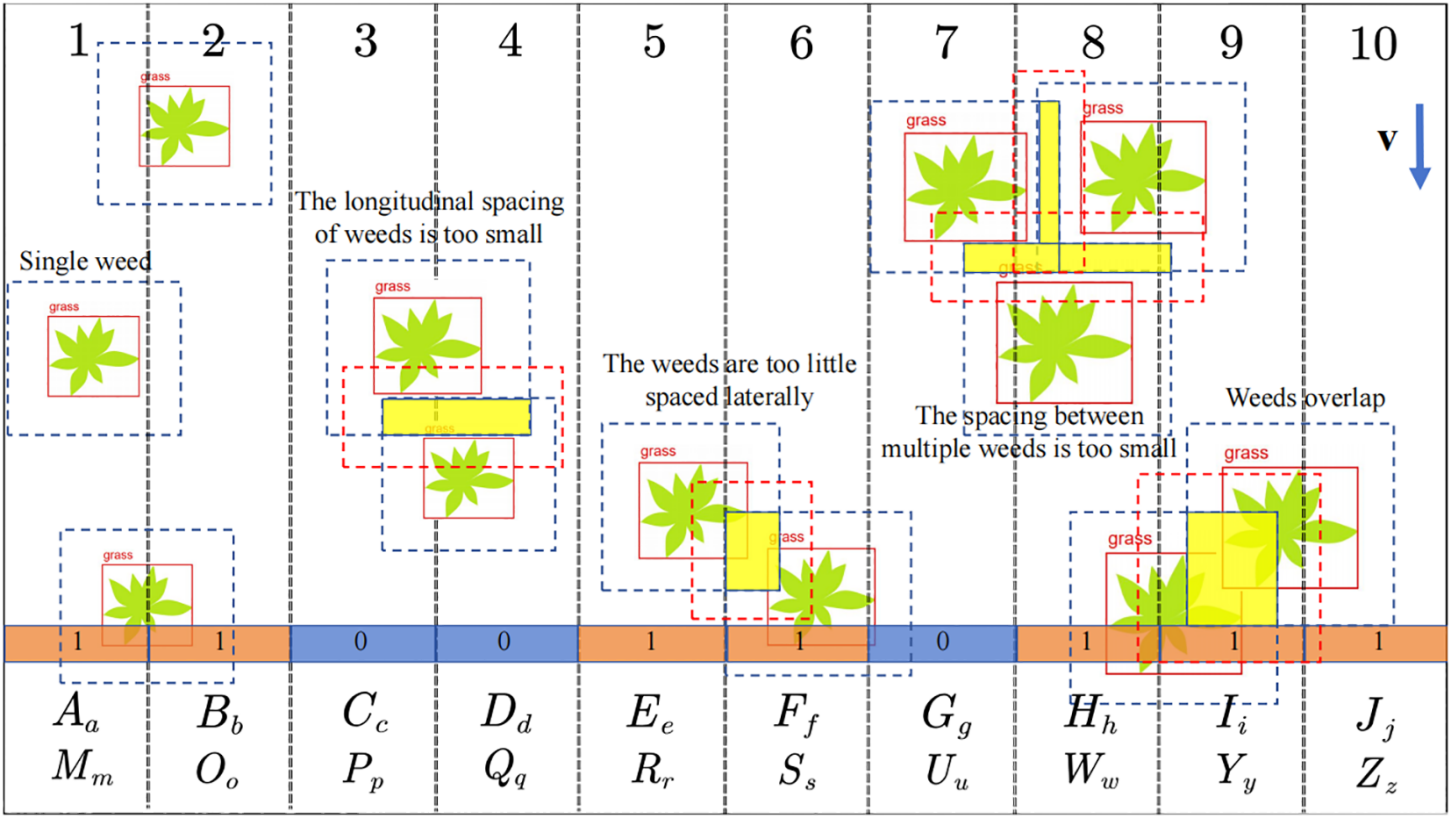
Schematic diagram of decision making for target spraying.

After the Yolov5-SGS completed the weed identification, the target spraying decision-making algorithm was used to differentiate the weeds with different distributions. The target spraying decision algorithm is shown in **Figure 5**.

Here the numbers 1 to 10 represent the serial numbers of the corresponding nozzles and the arrays [*X*] are the parameters in the hysteresis model.


$$\left[X\right]=\left[\begin{array}{cccc} \begin{array}{cc} A_{\text{a}} & B_{b}\\ M_{m} & O_{o} \end{array} & \begin{array}{cc} C_{c} & D_{d}\\ P_{p} & Q_{q} \end{array} & \begin{array}{cc} E_{e} & F_{f}\\ R_{r} & S_{s} \end{array} & \begin{array}{cc} \begin{array}{cc} G_{g} & H_{h}\\ U_{u} & W_{w} \end{array} & \begin{array}{cc} I_{i} & J_{j}\\ Y_{y} & Z_{z} \end{array} \end{array} \end{array}\right]$$

First, the image captured by the camera was divided into ten regions, as shown by the grid divided by the black double dashed lines in the figure, with the length of each grid corresponding to the spraying range of its nozzle. When the sprayer works, the image moves from top to bottom and Yolov5-SGS generates a real-time red solid line prediction box for weeds.

Secondly, for the case of a single weed, when the red solid line prediction box touches the grid, the corresponding area of the grid will change from 0 to 1. At the same time, as long as there is a red solid line prediction box in the grid, the judgment value will remain at 1 until all the red solid line prediction boxes have left the grid, and then the judgment value will change from 1 to 0.

In addition, for the distribution of multiple weeds, there are four scenarios: weeds too close together vertically, weeds too close together horizontally, multiple weeds too close together, and weeds overlapping. The blue dashed box is the minimum range of multi-weed spacing that the solenoid valve can respond to spraying. When the blue dotted line box of multiple weeds intersects, the intersection will become the yellow area shown in the figure, and will automatically generate a red dotted line box on the yellow area according to the minimum distance between multiple weeds that the solenoid valve can respond to spraying. At this time, the system will be generated according to the red solid line box and the red dashed line box for determination, when the red solid line box touches the grid, the corresponding grid will change from 0 to 1, the middle of the red dashed line box will make the grid to keep the determination of the results of 1, until all the red solid line box away from the grid determination will change from 1 to 0. That is to say, there are neighboring multi-weed grid, the algorithm will be for the phenomenon so that the solenoid valve to remain on continuously.

#### 2.4.2 Algorithm for target spraying hysteresis

**Figure 6:**
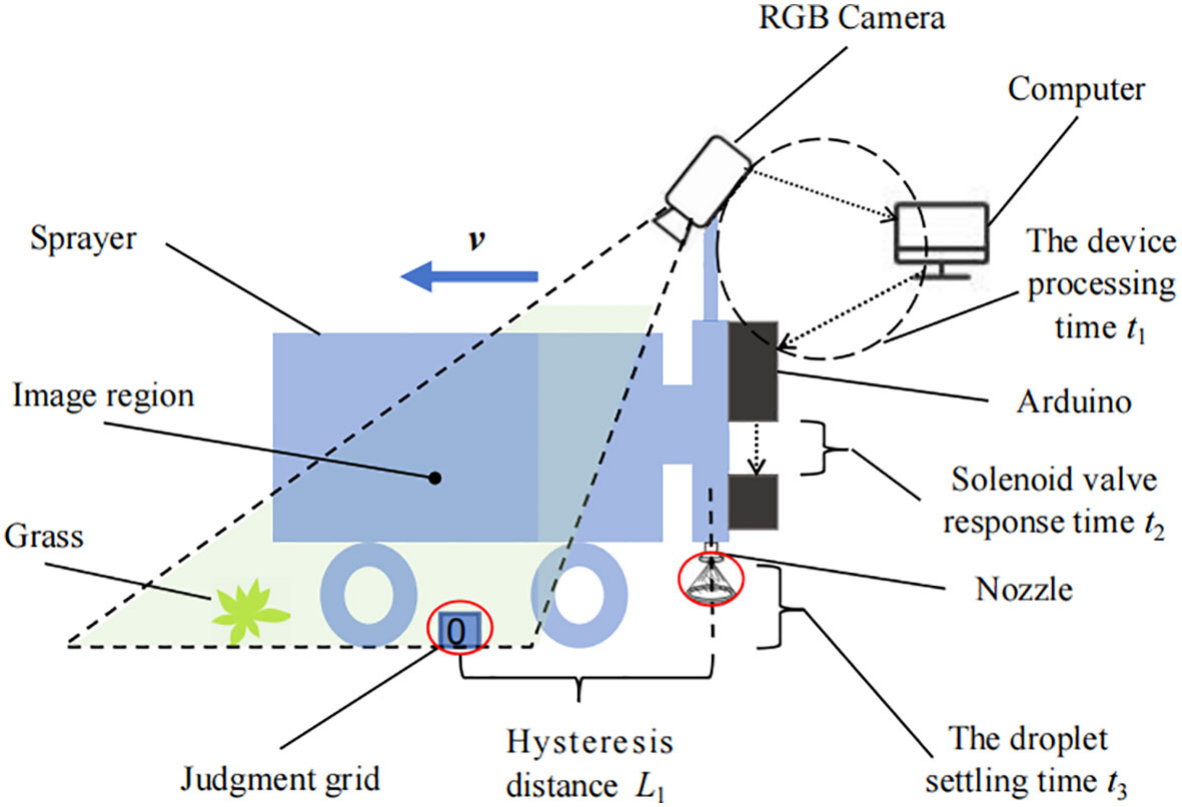
Schematic diagram of weed hysteresis to target spraying.

There is a distance *L*_1_ between the position of the judgment grid for the target decision and the spray nozzle, which requires the sprayer to travel this distance before controlling the solenoid valve for spraying. And in the process of sending the spraying command, there is a delay in the hardware system, and there is a delay time for the liquid to float down to the weeds. The schematic diagram is shown in **Figure 6**.

Referring to **Figures 5** and **6**, taking nozzle No. 1 as an example, *v* is the traveling speed of the sprayer. The delay time *t*_c_ includes the equipment processing time *t*_1_, the solenoid valve response time *t*_2_, and the liquid droplet settling time *t*_3_. When the grid determination value changes, the position *A*_a_ of the spray nozzle opening and closing includes: the distance between the determination grid position and the spray nozzle *L*_1_. The spray machine traveling distance *L*_2_ at the delay time *t*_c_. In order to ensure the target hit rate and spraying rate, as well as to reduce the error caused by the speed measurement hardware, the distance *L*_3_ is compensated at the above distance.


$$t_{c}=t_{1}+t_{2}+t_{3}$$

$$L_{2}=vt_{c}$$

$$A_{a}=L_{1}+L_{2}+L_{3}$$

The GNSS antenna and the motor speed measuring device feedback the speed at a frequency of 10 Hz, these speed data are discrete, so it is not accurate to calculate them by multiplying the speed by the time and then adding them up, here they are calculated by numerical integration. Numerical integration methods include the Simpson method and the trapezoidal method. In general, the Simpson method is more accurate than the trapezoidal method because it uses a more sophisticated curve approximation method that takes into account the quadratic polynomial relationship between the velocity data points. As a result, the Simpson method typically provides a more accurate estimate of the integral. The trapezoidal method is actually a special case of the Simpson method, which assumes that the relationship between the velocity data points is linear. In order to make the value of distance Mm more accurate, the composite product formula method is used, which consists of the composite Simpson formula and the composite trapezoidal formula. In the case where the speed is essentially uniform and is measured with a feedback of 0.1 s, the difference between the two methods of integration may not be very significant, and the spray vehicle is essentially moving at a uniform speed during spraying, with a relatively small change in speed, which makes the error between the two methods relatively small. The error of the two methods is relatively small. However, the composite Simpson method requires that the data sub-interval must be even, in the process of the actual working condition of the sprayer, the entire sprayer travel time according to the 0.1 seconds divided by the parity of the interval is uncertain, so the composite trapezoidal method is used for calculation.


$$h=\frac{t}{n}=0.1$$

$$t_{k}=kh=0.1k(k=0,1,2,\ldots ,n)$$

$$\begin{array}{c} M_{m}=\int_{0}^{t}v(t)dt\approx \frac{h}{2}\left[v(0)+v(t_{k})+2\sum_{k=1}^{n-1}v(t_{k})\right]\\ =0.05\left[v(0)+v(t_{k})+2\sum_{k=1}^{n-1}v(t_{k})\right] \end{array}$$

Here *h* is the step size. *n* is the number of times the velocity is recorded, with an initial value of 0. The distance traveled by the sprayer after a change in the grid determination value (when 0 becomes 1 or 1 becomes 0) is *M*_m_. The time is *t*. The initial values are all 0. *t*_k_ is the *k*th time value, which is a multiple of 0.1. *v*(0) is the value of the velocity of the sprayer at the time of a change in the grid determination value. *v*(*t*_k_) is the *k*th GNSS antenna feedback velocity value.

Here after initialization of the system *a*=*m*=1, the controller reads the forward speed of the sprayer in real time, and when the value of the grid determination becomes 1, the controller calculates the positions *A*_a_ and *M*_m_ where the spraying starts and updates *A*_1_ and *M*_1_ in the array [*X*]. When *M*_1_≥*A*_1_, the corresponding nozzle starts spraying, shifts the subscripts of *A*_a_ and *M*_m_ back by one bit, *a*=*a*+1, *m*=*m*+1, and resets *t*_c_, *t*_k_, *k*, *A*_2_ and *M*_2_ to 0. When the value of the grid determination becomes 0, the controller calculates the position *A*_a_ and *M*_m_ of the end of the spraying, updating them in the array [*X*], and when *M*_2_≥*A*_2_, the nozzles end spraying, *a*=*a*+1, *z*=*z*+1, and reset *t*_c_, *t*_k_, *k*, *A*_3_ and *M*_3_ to 0.

**Figure 7:**
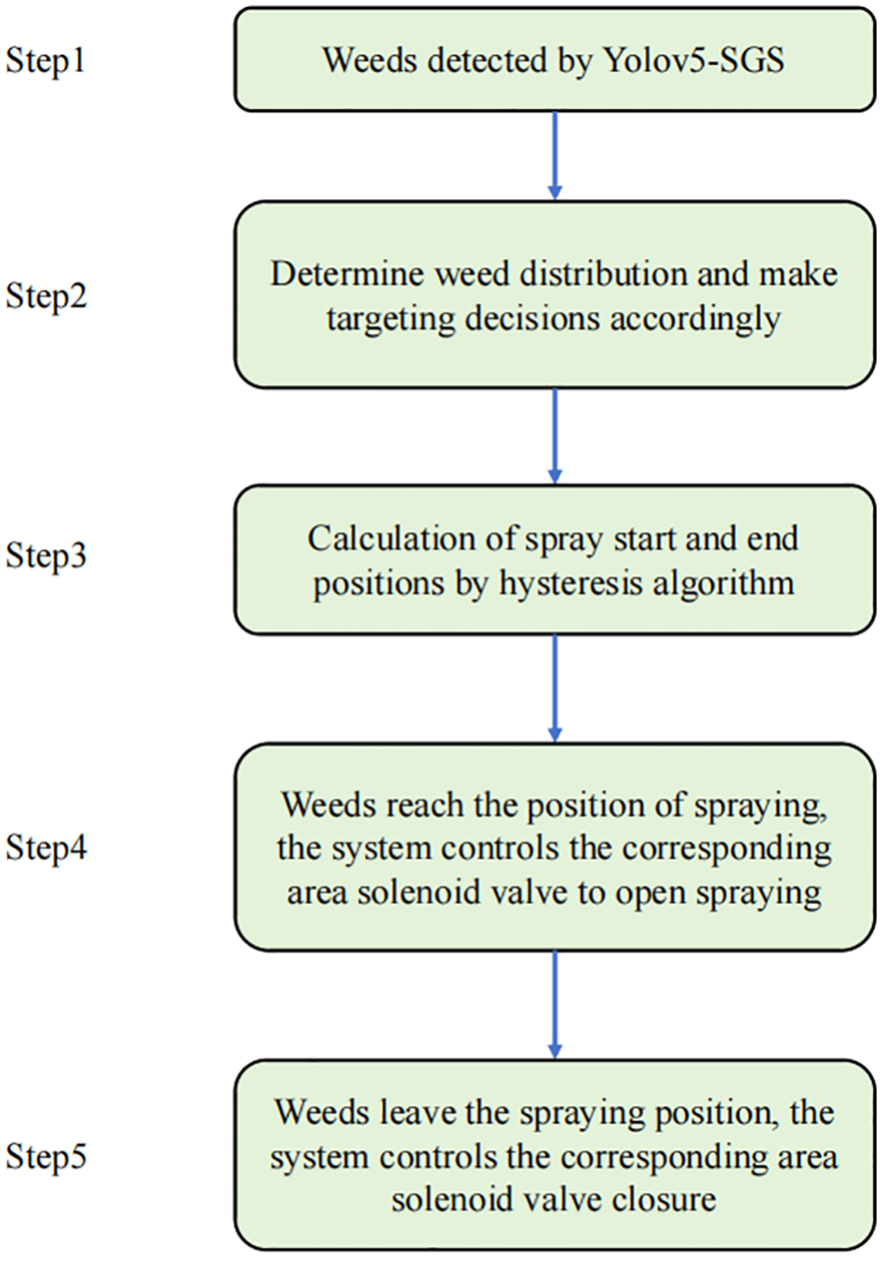
Algorithm workflow diagram.

In array [*X*], other groups of printhead parameters are updated in the same way as the first group. Simply put, the hysteresis model controls the corresponding printheads to turn on or off by determining whether the downstream parameters in the array [*X*] are greater than or equal to the upstream parameters of the corresponding position. Combined with the target decision-making algorithm, the entire algorithm workflow is shown in **Figure 7**.

## 3 Experiments

### 3.1 Experimental environment for model manipulation

As shown in **Table 2**, the computer selected for this study is Y9000X manufactured by Lenovo (Beijing, China), based on Intel(R) Core(TM) i7-12700H CPU, 16GB, NVIDIA GeForce RTX 3050Ti Laptop GPU hardware environment for weed model training and testing experiments, running on Python 3.8. The deep learning model is Pytorch 1.11.0+ Torchvision 0.12.0, and the running system is Windows 11.0. During the training process, the initial learning rate is set to 0.001, the ratio between the final learning rate and the initial learning rate is set to 0.2, the momentum of the SGD optimizer is set to 0.937, the number of rounds of the learning rate preheating is set to 3, the momentum at preheating is set to 0.8, and the biased learning at preheating is set to 0.1. rate was set to 0.1, and a cosine annealing learning rate tuning strategy was used. The model was trained for a total of 300 epochs.

**Table 2:** Training platform configuration details.

Enterprise	Configuration Details
CPU	12th Gen Intel(R) Core(TM) i7-12700H@2.3GHZ
GPU	NVIDIA GeForce RTX 3050 Ti Laptop GPU
Development environment	Python 3.8
Deep learning Module	Pytorch1.11.0+Torchvision 0.12.0
Operating system	Windows 11.0

### 3.2 Model detection effectiveness analysis

**Figure 8:**
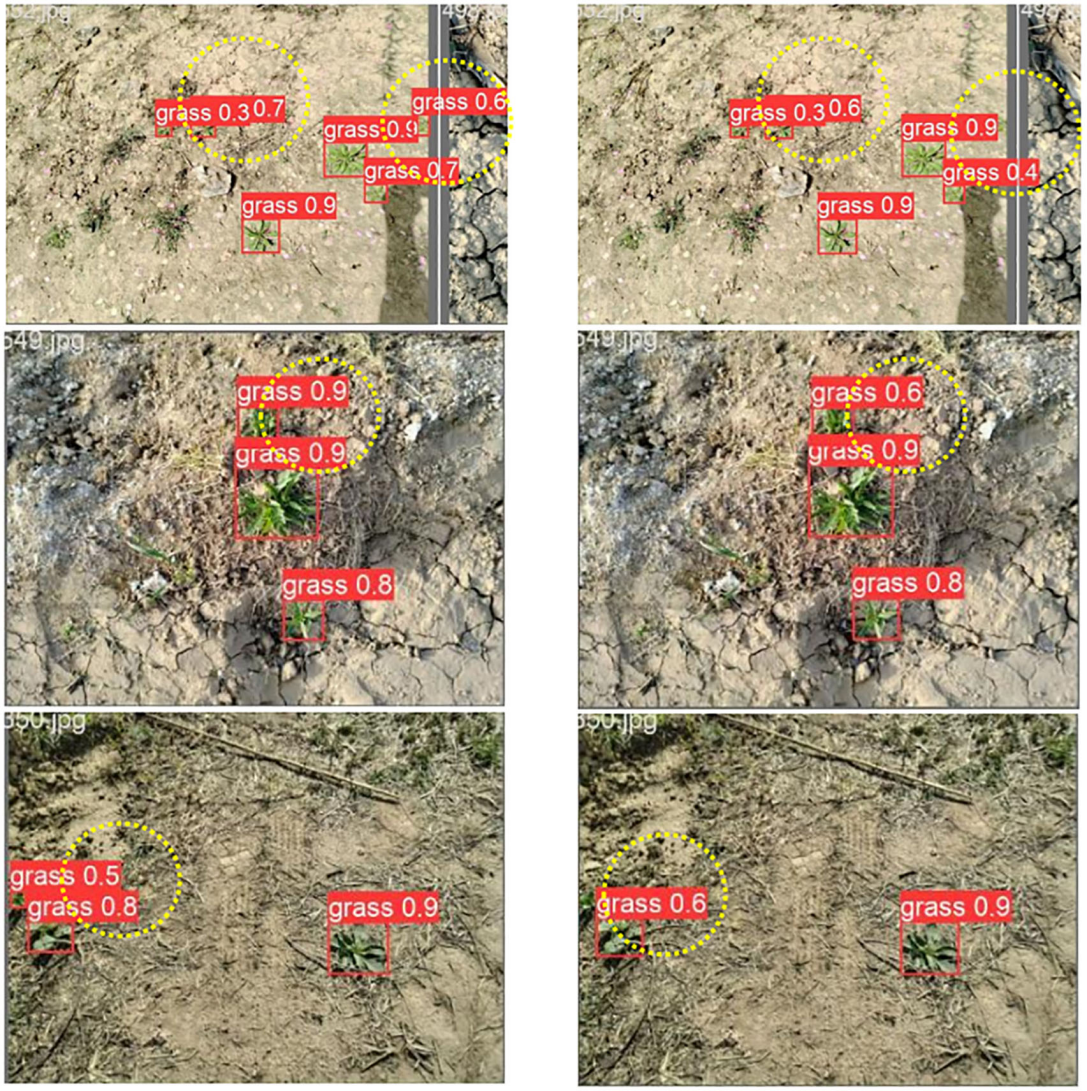
Comparison of detection results: the left side is the identification result of Yolov5-SGS, and the right side is the identification result of Yolov5s.

The improved weed detection results of Yolov5-SGS and Yolov5s are shown in **Figure 8**, in which the left side is the detection result of Yolov5-SGS and the right side is the detection result of Yolov5s. Through the comparison in the figure, Yolov5-SGS is more accurate in weed target detection, which can effectively solve the problems of small target unrecognizable, low precision of multi-target coexistence detection and inaccurate multi-target cross detection. Due to the wide shooting range, some weeds are smaller in the image and Yolov5s didn’t recognize these small weeds, while the improved Yolov5-SGS can detect these small weeds and the detection effect of multi-weed coexistence rises. Moreover, Yolov5-SGS has a small detection leakage and improves the accuracy of detection when cross-detecting multiple weeds. This is due to the addition of the SimAM attention mechanism expanding the distinction between weeds and background.

### 3.3 Ablation experiments

To evaluate the performance of each module, ablation and comparison experiments were conducted. Training and testing were performed on the same dataset and training parameters and the results are shown in **Table 3**.

**Table 3:** Results of ablation experiments with different optimization modules.

Modules	Yolov5s	Ghost Module	Slim-Neck	SimAM	GFLOPs	Model size (kB)	mAP (%)	F1 (%)
1	✓				15.9	14059	91.2	84.5
2	✓	✓			10.7	10324	90.2	83.4
3	✓	✓	✓		7.6	8083	90.5	84.9
4	✓	✓	✓	✓	7.6	8084	91.4	85.3

From the experimental results, it can be seen that: firstly, after optimizing the backbone feature extraction network using Ghost Module, the GFLOPs (computational complexity) and the model size decreased significantly, by 32.7% and 26.6%, respectively. Secondly, after using Slim -Neck to change the neck fusion network, at this time, F1 and mAP are improved by 1.5% and 0.3% compared with Yolov5s+Ghost Module, while the GFLOPs are decreased by 3.1, which indicates that the improved neck network improves the speed of the model in feature fusion and processing. Finally, introducing SimAM to adjust the attention distribution, F1 and mAP were improved by 0.4% and 0.9% compared to Yolov5s+Ghost Module+Slim-Neck, while GFLOPs remained unchanged, indicating that the self-attention mechanism improves the detection performance of small targets, widens the distinction between weeds and background, and does not complicate the model. The improved Yolov5-SGS model reduces the computational complexity and model size by half compared to the original model, and the accuracy of recognizing weeds rises.

### 3.4 Comparison of different target recognition models

The improved Yolov5-SGS network model is subjected to comparative experiments with many other Yolo series lightweight detection models to validate the performance of the proposed model. The experimental results are shown in **Table 4**.

**Table 4:** Comparison of models.

Models	GFLOPs	Model size (kB)	mAP (%)	F1 (%)
Yolov5-SGS	7.6	8084	91.4	85.3
Yolov4 tiny	5.7	6063	70.2	57.2
Yolov5s	15.9	14059	91.2	84.5
Yolov7 tiny	13.6	11984	91.3	85.2
Yolov8s	28.6	21977	90.0	83.7

The comparison shows that the improved model Yolov5-SGS has the smallest GFLOPs (computational complexity) and model size and has the highest mAP and F1 inside all the models compared to Yolov5s, Yolov7tiny, and Yolov8s. Although the GFLOPs and model size of Yolov5-SGS are not as small as Yolov4 tiny, the mAP and F1 of Yolov5-SGS are much higher than that of Yolov4 tiny, which indicates that YOLOv5-SGS has the advantages of low computational complexity and the ability to maintain a very high level of accuracy with a very small model, which are not found in other lightweight YOLO models. accuracy when the model is very small.

## 4 Experimental results and analysis

### 4.1 Experimental site

**Figure 9:**
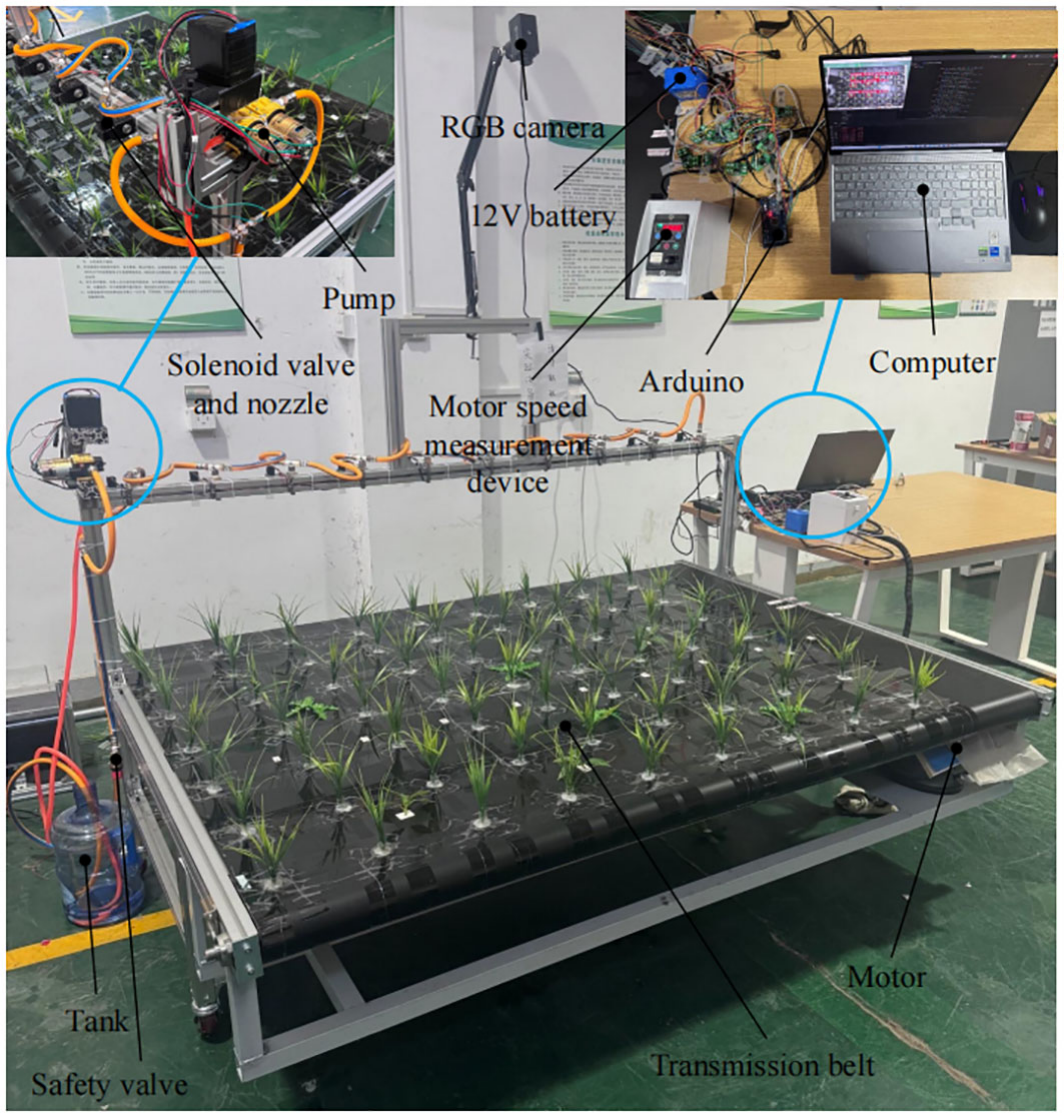
Field test of target spraying system.

To verify the accuracy of the target spraying decision and hysteresis algorithms and to evaluate the performance of the target spraying control system, a single target spraying control system test bed was built for testing. The test bed is shown in **Figure 9**. Here, the motor speed of the conveyor belt is sent to the computer in real time and converted into velocity values to replace the GNSS antenna for velocity measurements.

Simulated weeds and simulated wheat similar to those in the field were used in the experiment instead of real plants, and 600 images were taken as a dataset. The modified Yolov5-SGS network model was used in the same operating environment as before for 300 epochs of training. The trained model performed as follows in terms of accuracy metrics: 96.6% for P, 97.7% for R, and 99.1% for mAP.

### 4.2 System-to-target spraying effects

The camera and spray nozzle were fixed to the frame. At the beginning of the test, the simulated plants on the conveyor belt moved towards the back of the camera at randomly varying speeds *v* within the set speed intervals to simulate the actual situation of the sprayer traveling in the field. The speed intervals were set at 0.3-0.4 m/s, 0.4-0.5 m/s and 0.5-0.6 m/s. In each test, a water-sensitive paper was placed next to the simulated weed in order to provide an objective assessment of the spraying effect. This water-sensitive paper would quickly turn red upon contact with water, so that the actual effect of spraying could be clearly observed. In order to ensure the reliability and accuracy of the results, up to 50 trials were conducted by constantly changing the position and type of the simulated weeds on the conveyor belt. The number of weeds used in each test was 20, so that the total number of weeds tested in the 50 tests amounted to 1,000.

Recognition rate, spraying rate and hit rate were calculated based on the reddening of the water sensitive paper color, respectively. Recognition rate is the ratio of the number of weeds detected by the system to the number of all weeds. Spraying rate is the ratio of the number of weeds whose water-sensitive paper turns red to the number of detected weeds. The hit rate is the product of the recognition rate and the spraying rate. Specific test results are shown in **Table 5**.

**Table 5:** Spraying results of different speed intervals for the target spraying system.

Speed range (m/s)	Recognition rate (%)	Spraying rate (%)	Hit rate (%)
0.3~0.4	98.5	99.8	98.3
0.4~0.5	98.1	98.2	96.3
0.5~0.6	97.4	95.7	93.2

The extent of weed coverage by the system spray is recorded by the length of the water-sensitive paper that turns red. Coverage was the ratio of the length of the weed to the length of the water-sensitive paper discoloration. The targeting error is the error between the center position of the weed and the center position of the water-sensitive paper discoloration, and the mean absolute error and root mean square error were calculated. The results are shown in **Table 6**.

**Table 6:** Results of spray coverage.

Speed range (m/s)	Coverage rate (%)	Mean absolute error (cm)	Root mean square error (cm)
0.3~0.4	94.3	1.5	2.6
0.4~0.5	93.7	2.1	3.6
0.5~0.6	87.8	3.8	6.6

According to the experimental results, in the velocity interval of 0.3~0.4m/s and 0.4~0.5m/s, the spray system has a very good performance in the rate of target spraying and the coverage of spraying on weeds, especially in the realization of target spraying on multiple weeds in close proximity to each other, both of them can ensure that the mist adheres to the mist, and the spraying rate of the whole experiment reaches 99.8% and 98.2%, respectively, which indicates that the target spraying decision and the hysteresis algorithm is effective. However, there was a substantial decrease in the precision of the target spraying at 0.5 to 0.6 m/s, which was due to the inability of the velocimetry device to feed back the speed at a higher frequency, leading to errors in the calculation of the weed position by the target spraying decision and hysteresis algorithms. Therefore, at faster speeds, a velocimetry hardware device with a higher feedback frequency is needed to ensure the accuracy of the algorithm.

### 4.3 Analyses and discussions

Current weed detection and smart spraying systems still face many bottlenecks, and the processing power of computers and the costs associated with system hardware limit the development of target spraying systems (Vijayakumar et al., 2023). Fan et al. (2023) developed a weed detection and target spraying robotic system for seedling stage in a cotton field based on deep learning, which utilized three detection cameras to to acquire cotton and weed images of row plants in real time, each camera controls one spray nozzle each, and the three nozzles are responsible for spraying weeds in the corresponding area.Liu et al. (2021), on the other hand, deployed the DCNN model on a computer and used three webcams to perform weed identification with solenoid valves below each camera with a response time of less than 60 ms.Balabantaray et al. (2024) developed a weeding robot based on YOLOv7. The computer mainframe used by the robot for weed recognition was a Dell Precision 7,670 mobile workstation (12th Gen Intel Core i9-12950HX, 32GB RAM, NVidia RTX A4500 16 GB)), and the spraying system was equipped with a a single spray nozzle to target spray a single row area. These studies set a precedent for deep learning-based weed target spraying and also provide a solid foundation for target weed control in terms of system design. The focus of this research is to achieve higher spraying accuracy on less costly hardware and to allow for a wider coverage of the target spraying work area. Therefore, we lightened YOLOv5s and ensured that the accuracy of the model rose, where the GFOLPs (computational complexity) and model size were reduced by 52.2% and 42.4%, respectively, while the mAP and F1 values were increased by 0.2% and 0.8%, respectively, so that the weed identification model could be deployed on computers with limited processing power. In addition, the choice of a wide-angle distortion-free RGB camera to cover more area reduces the need for multiple cameras, and a rational structural arrangement allows identification of field areas up to 2m in width, thus further reducing the associated costs. In terms of target localization, Li He et al. (2022) combined deep learning algorithms with spraying technology to design a machine vision accurate real-time targeted spraying system for field scenarios, and proposed a solenoid group switching grid decision algorithm for single weeds.Zhao et al. (2022) developed a machine learning-based targeted spraying system equipped with an algorithm can differentiate between cabbages and weeds and accurately spray herbicides on single cabbages to resist the threat of pests and diseases.The algorithm designed in this study is very effective in localizing individual plants with large spacing and canopy and proposes a solution for the hysteresis problem in the operation process. Upadhyay et al. (2024) designed a spraying system based on machine vision by placing cameras in the three sets of grids in the vertical direction of the recognized area, which effectively improved the problems of single weed plant localization and missed detection. The above study provides an excellent reference solution for plant localization, which has a significant impact on the key operational aspects of target spraying. In this study, considering the low response frequency of most solenoid valves on the market, an algorithm for target spraying decision and hysteresis control is designed based on deep learning, which is capable of distinguishing the different distributions of single-plant and multi-plant weeds. After completing weed identification, the system accurately sprays weeds with different distributions by controlling the solenoid valves, thus avoiding the problem that the solenoid valves cannot respond in time when multiple weeds are too close together. This approach eliminates the need for farmers to purchase expensive high-frequency response solenoid valves, thus controlling the cost while performing precise weed control by carrying this weed localization and recognition algorithm. To address the problem of hysteresis characteristics on the hardware side, this study innovatively proposes to utilize the numerical integration method to calculate the speed in order to cope with the challenge that the data fed back from the speed measuring device is discrete, which effectively solves the hysteresis problem between the spraying hardware.

It is important to note that the targeted spraying system developed in this study is still in the bench stage. The motor-controlled weed movement speed is easier to control on the experimental bench compared to the complex field environment. At the same time, the experimental bench can more easily evaluate the recognition effect of the algorithm and the spray response performance compared to the influence of multiple factors in the field. Therefore, the spraying data we collected on the experimental bench will provide important support for the integration of targeted spraying systems in sprayers. Although there are some limitations in the experimental bench test, its modular design allows multiple targeted spraying systems to be carried in the spraying machine, and based on the shared speed measurement module and herbicide supply module, the image acquisition module and spray execution module are added to meet the actual spraying requirements. In real scenario experiments, problems such as inaccurate weed identification, fog liquid offset and sprayer speed measurement need to be considered. In the future, we plan to integrate dual GNSS antennas for RTK differential speed measurement in the sprayer’s target spraying system, and equip it with a high-frame-rate anti-shake RGB camera and a high-performance on-board computer. These high-performance hardware will significantly reduce the weed recognition time and system latency, and reduce the image jitter due to uneven road surface, thus improving the weed recognition rate.

## 5 Conclusion

In this paper, a deep learning-based weed detection and target spraying control method for wheat field at tillering stage is designed and deployed on the target spraying control system testbed. The study draws the following conclusions: firstly, a target spraying control system is proposed, including an image acquisition and real-time speed measurement module, a signal conversion module, a spray execution module, and a herbicide supply module. Second, in order to reduce the computational complexity and model size while guaranteeing the recognition accuracy, the backbone and neck networks of Yolov5s are lightweighted and improved, and a self-attention mechanism is introduced. These improvements resulted in a reduction of 52.2% and 42.4% in the model’s GFOLPs (computational complexity) and model size, respectively, and an increase of 0.2% in the mAP value and and 0.8% in the F1 value, which provided the best performance in comparison with other YOLO series lightweight models. Finally, to address the problem of inaccurate spraying due to the close proximity of different weeds and the hysteresis characteristics of the target spraying hardware, we designed an target spraying decision and hysteresis algorithm and deployed the integrated algorithm on a test bed for experimental validation. The experimental results show that the spraying rate reaches 99.8% and 98.2% in the velocity intervals of 0.3~0.4 m/s and 0.4~0.5 m/s, and the coverage rate is 94.3% and 93.7%, respectively. This indicates that the target spraying system differentiated the spraying of weeds with different distributions and the problem of hysteresis between the hardware was effectively solved. However, at faster speed intervals of 0.4 to 0.5 m/s, the spraying and coverage rates decreased to 95.7% and 87.8%, respectively, and thus a velocimetry device with higher feedback frequency is required to ensure the accuracy of the algorithm at faster speeds.

## Data Availability

The raw data supporting the conclusions of this article will be made available by the authors, without undue reservation.
